# Contribution of Governance and Socioeconomic Factors to the *P. aeruginosa* MDR in Europe

**DOI:** 10.3390/antibiotics11020212

**Published:** 2022-02-08

**Authors:** Julián Riaño-Moreno, Jhoana P. Romero-Leiton, Kernel Prieto

**Affiliations:** 1 Faculty of Medicine, Cooperative University of Colombia, Villavicencio 500002, Colombia; julian.camilo.riano@gmail.com; 2 Department of Bioethics, El Bosque University, Bogota 110111, Colombia; 3 Faculty of Engineering, Cesmag University, Pasto 520001, Colombia; 4 Georgian College, Barrie, ON L4M 3X9, Canada; kernel@ciencias.unam.mx

**Keywords:** governance index, data panel, machine learning, corruption index, multi-drug resistance, 92D30, 92D25, 49J15, 34D20, 62XX62, 62PXX

## Abstract

This work aims to explain the behavior of the multi-drug resistance (MDR) percentage of *Pseudomonas aeruginosa* in Europe, through multivariate statistical analysis and machine learning validation, using data from the European Antimicrobial Resistance Surveillance System, the World Health Organization, and the World Bank. We ran a multidimensional data panel regression analysis and used machine learning techniques to validate a pooling panel data case. The results of our analysis showed that the most important variables explaining the MDR phenomena across European countries are governance variables, such as corruption control and the rule of law. The models proposed in this study showed the complexity of the antibiotic drugs resistance problem. The efforts controlling MDR *P. aeruginosa*, as a well-known Healthcare-Associated Infection (HCAI), should be focused on solving national governance problems that impact resource distribution, in addition to individual guidelines, such as promoting the appropriate use of antibiotics.

## 1. Introduction

Antimicrobial resistance (AMR) is an ever-growing concern in medicine and public health globally. Patients infected by AMR bacteria require extended hospital stays and costly and multiple treatments that result in an economic impact on both the patients and the healthcare system [1].

Several pathogens have started to develop AMR, particularly that to first-line, inexpensive, broad-spectrum antibiotics, while the introduction of new drugs (e.g., fluoroquinolones) has been followed by the emergence and dissemination of resistant strains [2,3]. 

Although single resistance is an important public health problem, MDR is a more critical and growing problem in the world. MDR tuberculosis caused 1.5 million deaths in 2018 (251,000 with HVI) [4]. In the United States (US), two of three deaths related to antibiotic-resistant pathogens are caused by MDR organisms commonly associated with healthcare [5].

*P. aeruginosa* [6,7] is a Gram-negative bacterium widely recognized as a microorganism related to HCAI. It is a ubiquitous environmental bacterium that causes opportunistic human infections, such as urinary tract infections, respiratory system infections, dermatitis, soft tissue infections, bacteremia, bone and joint infections, gastrointestinal infections, and a variety of systemic infections, particularly in patients with severe burns, and in cancer and AIDS patients who are immunosuppressed [6]. 

The eradication of *P. aeruginosa* has become increasingly difficult due to its remarkable capacity to resist antibiotics, which includes biofilm-mediated resistance and the formation of multidrug-tolerant persisted cells [8]. 

The European Antimicrobial Resistance Surveillance Network (EARS-Net) claims that *P. aeruginos* a remains one of the major causes of healthcare-associated infection in Europe because of its ubiquitous nature and potential virulence [9]. In 2019, EARS-Net reported that 30.8% of the *P. aeruginosa* isolates were resistant to at least 1 of the 5 antimicrobial groups under regular surveillance (piperacillin-tazobactam, ceftazidime, fluoroquinolones, aminoglycosides, and carbapenems) across the European Union (EU) and European Economic Area (EEA) countries and reported a 12.1% population-weighted mean resistance in 2015–2019 to at least 3 antimicrobial groups [9] or MDR *P. aeruginosa* [10].

MDR *P. aeruginosa* (or MDR-Pa) is an important determinant of a higher rate of intensive care unit (ICU) admission and hospital mortality [11] and an increase in patient morbidity due to a higher incidence of surgery and longer duration of hospital stays [10]. Despite the enormous impact of MDR-Pa, the resistant mechanism is a complex and not fully understood phenomenon; several studies have described some of these mechanisms, which include well-described molecular phenomena, such as antibiotic-mediated selection, horizontal gene transfer, and others [12], even though there are very few models that study the factors favoring MDR over simple AMR, in general [13]. 

Although MDR and AMR phenomena occur in the molecular scenario, those mechanisms may be fostered and pushed by different social and behavioral determinants. Several studies have found that the general and imprudent consumption of antimicrobials, antimicrobial misuse, and overuse in hospitals—when inappropriate initial antibiotic therapy is prescribed—are the main causes for resistance [12,13,14]. However, some factors have been suggested as conditional factors to those well known in clinical settings, such as socioeconomic and political factors across countries, mainly those related to governance and regulation, which are usually poorly understood and minimally taken into account as leading factors in MDR and AMR phenomena [15].

Multiple studies have observed a pattern of AMR distribution across European countries, showing greater resistance in southern and eastern countries and lower resistance in northern and western countries. Researchers have suggested that this phenomenon is related to in-hospital and out-of-hospital imprudent antibiotic use practices [16] and underdeveloped action plans against AMR in southeastern European countries [17]. 

Additionally, there is evidence of higher AMR rates in low-income and middle-income countries, in comparison with high-income countries [18,19,20]. Although this effect has been related to much lower community consumption of antibiotics in high-income countries than middle–low income countries [19], this also could be related to differences in national health policies, since the control of AMR is generally centralized in policies, with national initiatives and commitments [21].

The quality of government and socioeconomic development are key elements of people’s well-being and health. Thus, these indicators have been a priority among EU policy makers for decades. However, there are remarkable differences in socioeconomic and governance performances across political communities over time in EU/EAA [22]: those differences are consistent with the AMR patterns in Europe. Stronger governance performance has been seen in northwestern over southeastern regions. The same differential pattern has been in socioeconomic performance. Additionally, there are important income differences between southeastern (lower income) and northwestern (higher income) EU/EAA countries [23]. 

Following these particular patterns across EU/EAA countries, we suggest that there are other non-consumption-related variables, such as the quality of governance, poverty, education, and community infrastructure, which affect health outcomes as structural determinants [15,20], also influencing MDR. In this context, we explore the MDR in *P. aeruginosa*, given that its resistance is less related to out-of-hospital care and antibiotic consumption per person, and more related to variables that could affect the institutional dynamics and the health system, in terms of resource distribution and national health policies. 

For this purpose, firstly we performed a clustering analysis to determine the countries across Europe that contribute the most to the MDR-Pa. We identified three clusters, and found that the two that contribute the most to the MDR-Pa are shaped mainly by countries from the southeastern region. 

Then, assuming the differences between these countries are mainly dependent on governance and socioeconomic factors, we ran a multidimensional data panel regression analysis for 30 EU/EAA countries from 2005 to 2018, including worldwide socioeconomic and governance indicators. Our results show that governance indicators are the variables that better fit our model in explaining the MDR-Pa variance across countries and over time. These results were validated by two machine learning methods (XGBoost and random forest). 

Through our models, we show the complexity of the antibiotic resistance problem. Geographical and temporal differences of MDR-Pa across EU/EEA countries could be explained by governance factors, such as corruption control, the rule of law, and other economic factors. Our findings provide another layer (macro-level) of factors for understanding MDR-Pa, including governance and socioeconomic factors, which are related to MDR-Pa. Thus, interventions focused on controlling MDR-Pa should be country-specific interventions; moreover, in addition to individual guidelines, such as promoting the appropriate use of antibiotics, these interventions should be focused on solving national governance problems that impact resource distribution and create health inequalities across countries, especially in southeastern countries. 

## 2. Materials and Methods 

All codes and source data used in this study can be found at the following Github link: retrieved on 5 July 2021 from https://github.com/jpatirom3/Governance_socioeconomic_resistance—for a detailed review 

### 2.1. Study Area

Europe contains around 50 countries, 27 of which are part of the European Union (EU), and some of the others are members of the European Economic Area (EEA). The EU/EEA is an economic and political union of 30 countries. It operates an internal (or single) market, which allows for the free movement of goods, capital, services, and people between member states [24]. A common geographical distribution of the EU/EEA countries into four regions: Northern Europe, Southern Europe, Eastern Europe, and Western Europe [25]. Table A1 (see Appendix A) shows the geographical distribution of the countries of the EU/EEA regions that were used in this study.

### 2.2. Data Collection

The data were collected from the European Antimicrobial Resistance Surveillance System (EARSS), the World Health Organization (WHO), and the World Bank. Their datasets are available through the European Centre for Disease Prevention and Control (ECDC) (https://www.ecdc.europa.eu/en, accessed on 5 July 2021), the World Health Organization (WHO) (https://www.who.int/health-topics/, accessed on 5 July 2021), and the Worldwide Governance Indicators (WGI) project (https://info.worldbank.org/governance/wgi/, accessed on 5 July 2021).

We used the EARSS dataset, corresponding to the resistance percentages and MDR percentages to *P. aeruginosa*. The original dataset contained information collected from 2000 to 2018, of 8 bacteria, namely: *Acinetobacter* spp.; *Enterococcus faecalis*; *Enterococcus faecium*; *Escherichia coli.*; *Klebsiella pneumoniae*; *Pseudomonas aeruginosa*; *Staphylococcus aureus*; *Streptococcus pneumoniae*. This dataset corresponded to the 30 EU/EEA countries.

For this study, MDR-Pa was defined as 1 isolate resistant to at least 3 antibiotic classes [10,26]; therefore, the data for *P. aeruginosa* reported by EARS was restricted to the percentage of resistance of combined resistance to at least 3 antibiotic groups, reported out of piperacillin/tazobactam, carbapenems, fluoroquinolones, ceftazidime, and aminoglycosides, per country, from 2005 to 2018.

Then, non-informative data was removed and the socioeconomic variables were incorporated; this was to classify the obtained regions of the European continent and match the data by country. After an exhaustive data curation and reorganization, 401 observations (for 30 geographical units: countries (*i_1–30_*) and 14 periods of time (*t_2005–2018_*)), 12 variables, 8 independent variables (GDP_total, GOV_effect, GDP_health, CTRL_corrup, Rule_law, Per_cap_US, Out_pocket_exp, HDI), 1 dependent variable (R_multi or MDR), a temporal variable (Year), and a cross-section variable (Country) were obtained. The region variable was included to classify the most affected countries by MDR-Pa. The final variables considered in this study are specified in Table 1. 

### 2.3. k-Means Clustering

Through a k-means clustering, we analyzed the correlation between the EU/EEA countries with respect to the resistance percentage to *P. aeruginosa*. This technique was applied to group the percentage of the resistance data, to identify how the countries are grouped in relation to the resistance percentages. The Mojena criterion [27] was used to determine the optimal number of clusters. Once the best number of clusters was determined, the k-means technique was applied to group the data.

### 2.4. Panel Data Analysis

The multidimensional data panel regression analysis was run in the software *EViews v12*. This approach provided us with observations on cross-section units (in this case: geographical unites = countries), i=1,2,I,N, over repeated time periods, t=1I...,T (in this case: years). Meanwhile, we found difficulty in obtaining information over time for the individual countries. Due to the amount of missing data, and in order to maintain the homogeneity of the data, a polynomial interpolation of the missing data was performed. 

However, it was not possible to make up for the lack of data from the EARSS dataset (only with the resistance percentage information), which forced the construction of unbalanced panel data (i.e., when there are missing elements that result in an incomplete data series for an individual, or individuals are absent in some years for a given variable), with i=30 and t ∈ [8,14], Slovakia being the country with the fewest periods (t=8), followed by Belgium (t=10), and with most of them (20) having periods of 14. According to Hsiao [28], although many statistical proposals are built from the consideration of balanced panels, most of the empirical studies and the data that can be used only enable unbalanced panels, such as the one presented in this study.

We used the two-way fixed effects method (TWFE), fixing cross-section units and periods. Thus, it was necessary to apply a transformation of the data to eliminate the unobserved heterogeneity, which allowed the fixed effects estimator to take the form of an ordinary least squares (OLS) estimator. Finally, we proposed the TWFE model for MDR percentage across EU countries included in our dataset.

### 2.5. Pooling Panel Data Analysis Using Machine Learning

For the machine learning (ML) validation, we used models such as those used in the references [29,30,31]. We used the *Scikit-learn* package in *Python 3.1.2* (Netherlands). Firstly, we ran a Hausman test and an F-test to choose our model (pooled OLS or fixed effects). We modeled the relationship between the input (independent) variables, $x_{1},\;x_{2},...,x_{n},$ or features, and the output (dependent) variable, y, or target variable, as a non-linear relationship with the polynomial of degree, m, called the complexity on the ML framework, on the variables $\;x_{1},\;x_{2},...,x_{n}$, as follows:

(1) $$y=d_{0}+d_{1}x_{1}+\ldots +d_{n}x_{n}+d_{n+1}x_{1}x_{2}+\ldots +d_{n+m_{1}}x_{1}^{m}+d_{n+m_{1}+1}x_{2}^{m}...d_{n+M}x_{n}^{m}$$

Then, we used a polynomial of degree m=3, 4, 5 of the Polynomial (1) and built the covariance matrix of these polynomial variables, $x_{1},...,x_{i}^{l1}x_{j}^{l2}x_{k}^{l3},...,x_{n}^{m},$ such that l1+l2+l3=m, with respect the target variable, y, and have selected the polynomial variables, $x_{i}$, the covariance value of which concerns the target variable, $\left(x_{i},y\right)\;$, which has a greater value than a threshold value, δ={0.3,0.5,0.6}. 

Next, we split our data, as follows: 80% was used as training data and 20% was used as testing data. We retrieved the results of the $R^{2}$ and $R_{adjusted}^{2}$ on the test data for polynomial degrees m=3,4,5 and the threshold values δ={0.3,0.5,0.6} were selected.

We also used different types of variable filters. We first used the low variance filter, which consists of eliminating the variables whose variance is less than a threshold value, η. Apart from the linear regression (LR) algorithm, we have used the k-nearest neighbors (kNN) and the decision tree (DT) algorithms. The hyperparameters for the KNN are k = 5 neighbors, and n = 5 for the decision trees.

Furthermore, we applied the k best variable selection (kBVS) and the recursive feature elimination (RFE) methods as other filter techniques of the polynomial features. The k best variable selection method selected the k best variables, based on the Fisher test. 

Finally, we used the XGBoost and the random forest algorithms as two more options for the pooling method, with the combination of the Shapley additive explanations (*SHAP*) package [32]. The latter package was used to formulate some plots which could better explain the models. We used the following hyperparameters for the XGBoost: η=0.3, max_dept=3, subsample=0.5, iterations=10,000.

## 3. Results

### 3.1. k-Means Clustering Analysis

**Figure 1:**
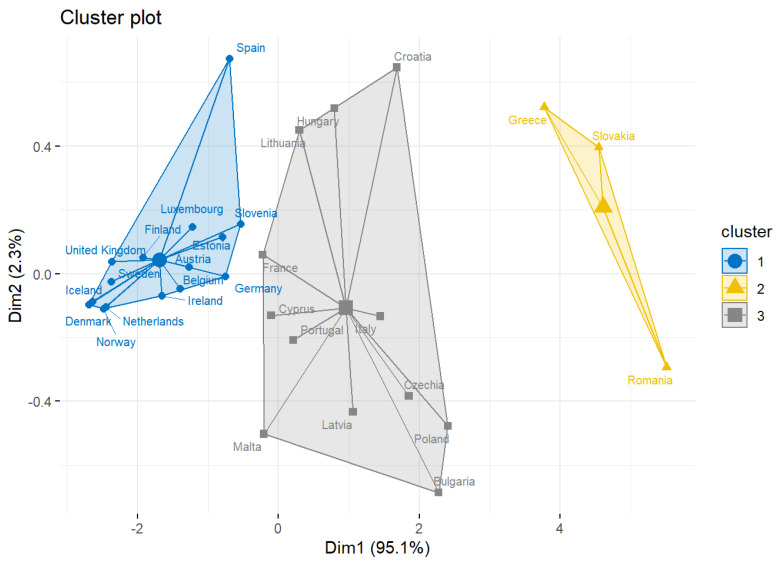
Clusters using k-means method.

Running the *k*-means classification method, we obtained three clusters, which are shown in Figure 1. Here, we observed that the Eastern European countries contribute the most in MDR-Pa in Greece, Slovakia, and Romania (Figure 1, cluster in yellow). Additionally, the second cluster (Figure 1, cluster in gray) that contributes to MDR-Pa is shaped mainly by countries in the eastern and southern regions of Europe (except for France). Finally, the countries that contribute the least are mainly from the northern and western regions of Europe (Figure 1, cluster 1 in blue). 

### 3.2. Panel data Analysis

Table A2 (see Appendix A) shows the results of the initial data panel analysis, before we removed those coefficients that were not significant (*p*-value > 0.05: this included GDP total per country, HDI, and GOV effect). Finally, the following model for MDR-Pa was obtained from the more significant variables’ coefficients, as given in Table 2:

(2) $$MDR-Pa=d_{1}Ctrl_{corrup}{}_{it}+d_{2}Rule_{law}{}_{it}+d_{3}OutPocketexp_{it_{}}+d_{4}PerCapUs_{it}+d_{5}Gdp\_health_{it}{}_{}+\mu_{i}+y_{t}+\varepsilon_{it}\;$$

Table A3 (see Appendix A) shows the results of the validation statistics for the final TWFE of the MDR-Pa model. In this sense, we could conclude that there is no residual autocorrelation (Durbin–Watson: *p*-value < 0.05), and reject the assumption of homoscedasticity (Breusch–Pagan: *p*-value < 0.05). On the other hand, the Jarque–Bera test for the residuals showed that they do not behave under the assumption of normality (*p*-value < 0.05). However, due to the number of observations, under a standard regression model, and subject to certain regularity conditions, the residuals will behave asymptotically normal. 

In Model (2), the most significant coefficients (p-value < 0.05), in their respective order of significance, corresponded to two governance variables (Rule_law and Ctrl_corrupt) and three health expenditure variables (GDP_health, Per_cap_US, and Out_pocket_exp). There is an inverse relationship between the MDR-Pa and the variables Ctrl_corrup, Gdp_health, and a positive relationship of the remaining variables with our model. The MDR-Pa model presented an $R^{2}=\sim 0.82$ (see Appendix: Table A4), which indicates that the variables obtained represent approximately 82% of the antibiotic MDR-Pa.

**Figure 2:**
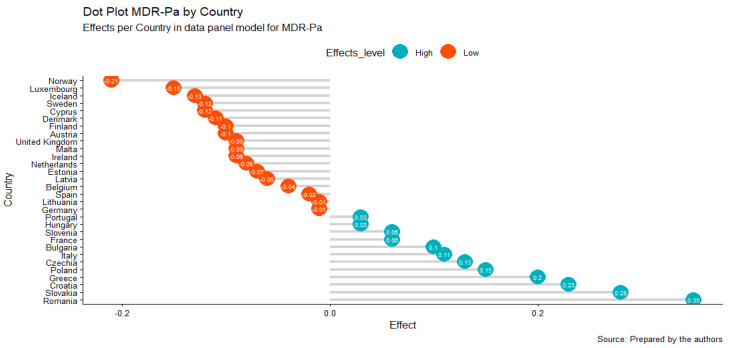
TWFE panel MDR-Pa model by geographical unit: country effects.

Additionally, we obtained effects for the geographic unit (Figure 2); these results were consistent with the results obtained in the cluster analysis, where it could be established that the countries that contribute the most to MDR-Pa are those found mainly in the southeastern region of Europe, particularly Greece, Slovakia, and Romania (Figure 1, cluster in yellow). Croatia was added through this model. The countries that contribute the least to antibiotic resistance are the northern (Norway, Iceland, Sweden, Denmark, and Finland) and the western countries (see Figure 2). It is striking how Cyprus, being a southeastern country, is one that contributes the least to MDR-Pa, in our model, and that France is a unique western country that contributes the most in the MDR-Pa phenomena in both the cluster analysis and data panel analysis. 

### 3.3. Pooling Panel Data Analysis Using Machine Learning

The results of the Hausman test and the F-test results are given in Table A5 and Table A6 (see Appendix A), respectively. Similar results are obtained using a Hausman test between the pooled OLS and the fixed-effects model. Since the F-test’s result showed that we must opt for a pooled OLS model, we opted to use ML without considering heterogeneity across time and countries.

The ML model was developed by applying the polynomial features technique given in Equation (1), using the XGBoost method and the random forest algorithm. Table A7 (see Appendix A) show the performance comparison of polynomial features and the threshold value, δ, concerning the covariance value, Cov(x_i,y), on the target variable, MDR-Pa. We could observe that the highest value of $R^{2}$ (~0.88 train, ~0.637 test, ESMR: 0.07) on the test data is obtained with the combination m = 3, δ = 0.3 for the output variable MDR-Pa.

Table A8 (see Appendix A) present the results obtained with the low variance filter. We observed that the highest $R^{2}$ value on the validation set was obtained with the combination of m = 3 degrees of the polynomial, algorithm set to LR, and *η* = 0.3 (selected features equal to 84) on the MDR-Pa variable

Furthermore, applying kBVS and RFE methods as filter techniques of the polynomial features, we obtained (Table A9) the highest $R^{2}$ value on the validation set, with 0.759 obtained with the k-best variable selection. 

Thus, the final model validation showed that both models, XGBoost and random forest, provide MDR-Pa. Highest $R^{2}\left(\sim 0.93\right)$ was provided by the training dataset XGBoost model (RMSE: 0.034), the testing dataset obtained the lower $R^{2}\left(\sim 0.77\;\right)$ and RMSE—highest at 0.063. The random forest MDR-Pa model obtained $R^{2}=\sim 0.80$ and RMSE: 0.055 (see Appendix A: Table A10). 

**Figure 3:**
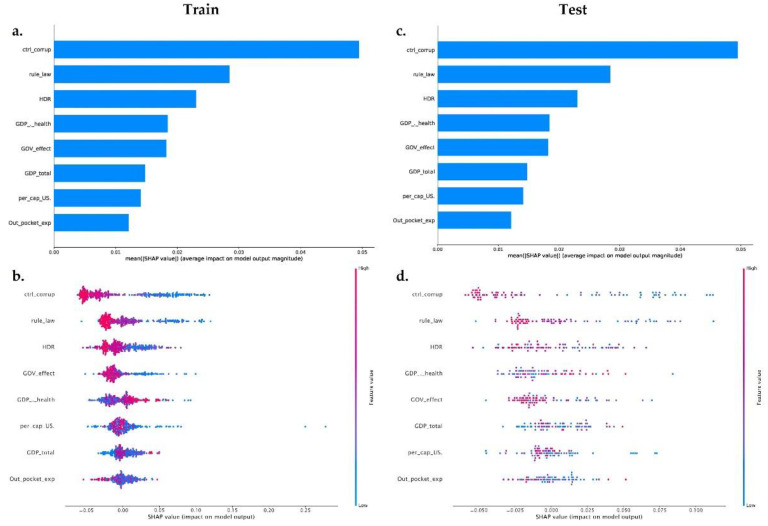
Machine learning using the XGBoost method. (**a**) Feature importance measured by SHAP values in the training dataset on the target variable MDR-Pa, respectively. (**c**) Feature importance measured by SHAP values in the testing dataset on the target variable MDR-Pa. (**b**–**d**) Impact of features for SHAP values for each feature for the XGBoost method in training (**b**) and testing (**d**) the dataset. Every observation is represented by one dot in each feature. The dot’s position on the x-axis represents the impact of that feature on the model’s prediction for the observation, and the dot’s color represents the value of that feature for the observation.

**Figure 4:**
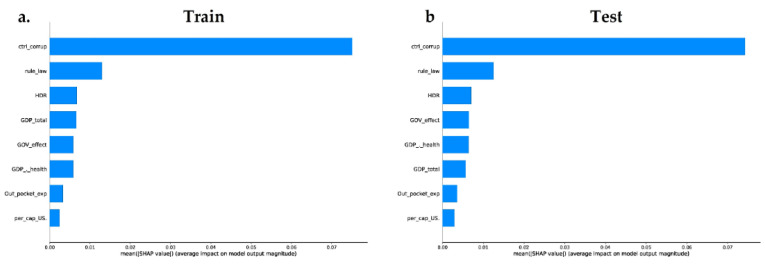
Machine learning using the random forest method. (**a**) Feature’s relevance/importance, measured by SHAP values of in the training dataset on the target variable MDR-Pa. (**b**) Feature importance measured by SHAP values in feature relevance of the testing dataset on the target variable MDR-Pa.

We trained a final model (on XGBoost and random forest algorithm) with all the governance and socioeconomic variables available in our dataset, to provide insight into the relative importance of each feature. We calculated the impact of the model output through the Shapley values (SHAP) for each feature. The XGboost model (Figure 3a,c) includes in the top features two governance variables (Ctrl_corruption and Rule_Law) and one socioeconomic variable (HDI)—the less important features in this model were Per_cap_US and Out_pocket_exp. The random forest model (Figure 4) shows the same behavior; however, the top feature in this model was a governance variable: Ctrl_corruption. 

Using the final XGboost model, we plotted SHAP for every observation across our dataset train and test dataset. In Figure 3b,d, each dot represents one observation, and the color represents the actual value of the feature from low values in blue to high values in red. The features are sorted by the mean of SHAP value. The positions on the x-axis represent the difference between prediction and observation—positive means the feature generates improvement in the prediction and negative corresponds to a worsened prediction.

The train and test dataset shows that the most important features are inversely proportional to the MDR-Pa, the countries with lower corruption index, rule of law index, or HDI index have a greater impact of MDR-Pa. On the other hand, Per_cap_US, Out_pocket_exp, GDP_total, show high and low values surrounding the mean; therefore, those are not interpretable results. Interestingly, the characteristics that have the most importance in the XGBoost and random forest models are the governance variables: especially Ctrl_corruption, which fits with the results of our data panel model. ML models differ from the data panel model, in that both show that HDI could be an important variable, explaining the variance of MDR-Pa across EU/EAA countries and across time. 

## 4. Discussion

*P. aeruginosa* is a well-known microorganism related to HCAI. In this context, control strategies based on the reasonable and adequate use of intra-hospital antibiotics, especially on patients in critical care, have been proposed. This problem has also led to extreme measures, such as the creation of new types of therapies or new antibiotics [33]. Here, we provided insights into the macro-level factors, across EU/EEA countries, related to MDR-Pa. To date, studies have been exclusively performed to identify the in-hospital factors [11,14] and the molecular mechanisms [28,34] related to MDR-Pa as strategies to understand this phenomenon. Our findings provide another layer of understanding both the transnational and temporal variances of MDR-Pa, defined by governance and socioeconomic variables, which work as possible conditional factors for appropriate institutional dynamics and adequate distribution of resources within the health systems across EU/EEA countries. 

Other authors [18,19,20,21,22,23] have seen the southeastern (higher)–northwestern (lower) pattern, especially as it is associated with AMR. Few studies have shown this effect in MDR. Gunther et al. [35] indicates that Eastern Europe shows a high incidence of multidrug-resistant Mycobacterium tuberculosis, with a low incidence in Western Europe. To the best of the authors’ knowledge, this is the first study applying clustering methods to reveal differences in MDR-Pa between southeastern and northwestern EU/EA countries. These results increase the evidence about how national differences in a community as large as the EU could be related to public health problems, such as MDR. Although these differences could be related to antibiotic usage between countries, as private practices carried out by providers and consumers [36,37], here, we moved forward, proposing that these dynamics are conditioned across countries and over time by macro-level factors, such as the government quality and the socioeconomic characteristics of the countries. 

In 2015, Collignon et al. [15] showed that factors other than antibiotic usage, such as the quality of governance and private health expenditures, are potentially very important in explaining the different levels of AMR seen in different EU countries. The authors include antibiotic usage as community consumption; however, *P. aeruginosa* is an HCAI-related microorganism—antibiotic resistance is poorly impacted by this factor. Here, we propose a clean model, validated by ML algorithms, with similar results for MDR-Pa, which avoid the community consumption effect. 

Control corruption was the most important variable explaining the MDR-Pa variance across countries and over time in our data panel model. Our model shows that, the lower the control corruption is in a country, the higher the MDR-Pa. ML models show that the control corruption explains most of the variation of MDR-Pa, with this indicator becoming the most important in the ML validation. Data panel and ML models exclude total GDP as an important variable explaining MDR-Pa; these results question the presumed relation between poverty and higher AMR [19].

On the other hand, our results show that gross domestic product for health (GDP_health) is an important factor. GDP for health has been shown to have a positive correlation with health outcomes [38]. In this context, the higher GDP for health in a country, the lesser the MDR-Pa. The corruption in a nation impacts the resource distribution for dealing with important health public issues, such as MDR-Pa. Factor and Kang [39], observing 133 countries, concluded that corruption is associated with lower levels of health expenditure. Thus, following our findings, the control of corruption is essential to guarantee the adequate distribution of the resources intended to cover the basic health needs of the population, so that, in a country where the control of corruption is lower, there is less probability of adequate spending on health to respond to problems, such as antimicrobial resistance, and there is also less confidence on the part of the population in their institutions.

Private health expenditure, such as current health expenditure per capita in the U.S. and out-of-pocket expenses, were included as part of the final data model as positively related factors. However, the XGBoost and random forest models show these variables are the less important features. High private health expenditure in a country suggests that healthcare is being delivered predominantly in the private sector, this means fewer controls and supervision. This impacts the health sector, resulting in fewer controls on antimicrobial distribution, time of drug therapy, and the volumes used [15]. This is true for community-level antimicrobial usage. Nevertheless, in the case of MDR-Pa, the ML algorithms results are expected, because MDR-Pa is mainly related to hospital-regulated factors. 

Interestingly, the rule of law (Rule_law) governance indicator, which evaluates the confidence in, and abides by, the rules of society, is negatively associated with MDR-Pa. The rule of law could be seen as a social determinant of health [40], in several ways, in a communitarian institution such as the EU/EAA. Implementing transnational health policies, improving skills of government health policymakers and providers, and the dissemination of information and experience across countries requires trust, not only in national institutions, but also in transnationals, such as the EU [41]. Here, we propose that countries with higher confidence in transnational institutions are more likely to follow the action plans against MDR, promoted by the EU [17,41]. Although there is a generally favorable view of the EU across EU countries [41], Greece, Czech Republic, Italy (southeastern countries), and France (western country), which are the countries that contribute the most in our MDR-Pa data panel model, have the largest proportion of unfavorable views of the EU. Even Italy, France, and Slovakia have deteriorated over time in their opinions of the EU. On the other hand, most of the northwestern countries (except France) had maintained or increased their favorability on the EU [42].

To our knowledge, this is the first study to include the human development index (HDI) as an integrated socioeconomic determinant to explain MDR. Strikingly, our data panel model excludes this variable, because we accept a *p*-value < 0.05 (it was significant for a *p*-value < 0.1 (0.0702)); however, the XGBoost model shows HDI as an important feature and inversely related MDR-Pa—that is, the higher human development in a country, the lower the MDR-Pa. This indicator includes three variables that must be taken into account: life expectancy at birth, schooling (expected years and mean years schooling), and GNI (gross national income) per capita. Since life expectancy at birth is an indirect health status, and quality of health is expected, in this context, MDR-Pa could be associated with lower quality health care systems, an association which has increased MDR threat to life expectancy, reducing neonatal survival in critical care settings [43,44]. On the other hand, increased use of emergency and hospitalization services due to lower health literacy [45] can increase the risk of infection for an MDR-Pa. Recently, Zhen et al. [46] found that GDP per capita is positively spatiotemporally related with antibiotic resistance in China, which, according to the authors, may be related to higher consumption of antibiotics. GNI per capita could be interpreted in the same way, but more studies are required to understand the importance of this variable. 

As far as we know, this is the first study that compares different multivariate methods (data panel and ML algorithms) to evaluate the impact of socioeconomic and governance indicators as macro-level factors explaining the MDR-Pa. Our findings increase the knowledge surrounding MDR, especially concerning such a rarely studied microorganism as *P. aeruginosa*. Additionally, this study provides another layer (macro-level) of factors in understanding MDR-Pa, including governance and socioeconomic factors explaining the variance of MDR-Pa across EU/EAA countries over time. 

Thus, interventions focused on controlling MDR-Pa should be country-specific; in addition to individual guidelines, such as those promoting the appropriate use of antibiotics, these interventions should be focused on solving national governance problems that impact resource distribution, and which also create health inequalities across countries, especially in southeastern countries.

A limitation of this study was the impossibility to include the in-hospital antibiotic consumption variable. European Surveillance of Antimicrobial Consumption Network (ESAC-Net) does not have complete data on the period and countries here studied. This hindered us from concluding that the governance and socioeconomic variables have a greater impact on the MDR than the antibiotic usage variable. It also reveals the need for better MDR surveillance systems in clinical settings to test this hypothesis.

We explored these patterns in exclusively EU/EAA countries because of the robust ESAC-Net surveillance system, and because it is a continent where good data are available from multiple countries. Since MDR is growing, it is necessary to be able to access better and better data to determine the factors related to MDR and to improve the antibiotic resistance surveillances systems, promoting action plans against this serious health public issue. Likewise, ML methods are becoming recursive, robust, and important technics to deal with the complexity of health problems. Here, we used two algorithms that revealed governance indicators as the most important determinants of MDR-Pa. However, the amount of data used (402 observations) could have restricted the power of the ML-based approximations; however, these results are supported by our data panel. This shows the strength of mixed methods in understanding and dealing with complex problems, such as MDR. 

## Figures and Tables

**Table 1:** Study variables: description.

Variable Name	Definition
R_multi (or MDR-Pa)	Antimicrobial multi-drug resistance MDR percentages. Defined as combined resistance to at least three antibiotics groups reported by EARSS out of piperacillin-tazobactam, ceftazidime, fluoroquinolones, aminoglycosides, and carbapenems.
Year	Years from 2005 to 2018 (time in data panel (*t_2005–2018_*))
Country	Country name (cross-section geographical units in data panel (*i_1–30_*))
Region	Region name (eastern, northern, southern, western)
GDP_total	Gross domestic product
GOV_effect	Government effectiveness index (−2.5 weak; 2.5 strong)
GDP_health	Gross domestic product for health
CTRL_corrup	Control of corruption index (2.5 weak; 2.5 strong)
Rule_law	Rule of law (2.5 weak; 2.5 strong)
Per_cap_US	Current health expenditure per capita in the US
Out_pocket_exp	Out-of-pocket expense
HDI	Human development index (0–1)

**Table 2:** Coefficients for the final panel data for the TWFE of the MDR-Pa model.

Variable	$\mathrm{Coef}\;(d_{\dot{l}})$	SE	p	CI 95%
Gdp_health	−0.018	0.0065	0.0061 ***	[−0.0311, −0.0052]
Ctrl_corrup	−0.079	0.0270	0.0035 ***	[−0.1325, −0.0263]
Rule law	0.1177	0.0317	0.0002 ***	[0.0552, 0.1801]
Per_cap_US	2.58 × 10^−5^	9.28 × 10^−6^	0.0056 ***	[7.60 × 10^−6^, 4.41 × 10^−5^]
Out_pocket_ exp	0.0053	0.0018	0.0052 ***	[0.0015, 0.009]

*** *p*-value < 0.05.

## Data Availability

The data were collected from the European Antimicrobial Resistance Surveillance System (EARSS), the World Health Organization (WHO), and the World Bank. Their datasets are available through the European Centre for Disease Prevention and Control (ECDC), the World Health Organization (WHO), and the Worldwide Governance Indicators (WGI) project.

## Appendix A

**Table A1:** Geographical distribution of the countries of the EU/EEA regions used in this study.

Region	Countries
Northern	Estonia, Finland, Ireland, Iceland, Norway, Sweden, United Kingdom, Lithuania, Latvia, Denmark.
Southern	Cyprus, Greece, Spain, Croatia, Malta, Slovenia, Italy, Portugal.
Eastern	Bulgaria, Czechia, Hungary, Poland, Romania, Slovakia.
Western	Austria, Germany, France, Luxembourg, Netherlands, Belgium.

**Table A2:** Coefficients for the initial panel data for the TWFE of the MDR-Pa model.

Variable	$\mathrm{Coef}\;(d_{\dot{l}})$	SE	p-Value	CI 95%
Gdp_health	−0.0259	0.0106	0.0149 ***	[−0.0468, −0.0468]
GDP_total	−1.07 × 10^−6^	1.59 × 10^−6^	0.5019	[−4.18 × 10^−6^, 2.05 × 10^−6^
HDI	−1.0664	0.5871	0.0702 *	[−2.2212, 0.0883]
GOV_effect	0.02323	0.0272	0.3936	[−0.0302,0.0767]
Ctrl_corrup	−0.0818	0.0278	0.0035 ***	[−0.1365, −0.0271]
Rule law	0.1139	0.034	0.0009 ***	[0.0468, 0.1809]
Per_cap_US	4.02 × 10^−5^	1.79 × 10^−6^	0.0249 ***	[5.09 × 10^−6^, 7.54 × 10^−5^
Out_pocket_ exp	0.006	0.0019	0.0023 ***	[0.0021, 0.0098]

*** *p*-value < 0.05, * *p*-value < 0.1.

**Table A3:** Two-way fixed effects (TWFE) of the final MDR model validation tests.

Test	Statistic	*p*-Value
Durbin–Watson	1.366244	0.0000 ***
Breusch–Pagan	649.0717	0.0000 ***
Jarque–Bera	822.4368	0.0000 ***

*** *p*-value < 0.05.

**Table A4:** $R^{2}$ and $R_{adjusted}^{2}$ for the final TWFE of the MDR-Pa model.

$R^{2}$	0.816533
$R_{adjusted}^{2}$	0.792106

**Table A5:** Hausman test between fixed and random effects.

$\xi^{2}$	DF	p-Value
96.50	9	2.2 × $10^{-16}\;$***

*** *p*-value < 0.05.

**Table A6:** F-test between pooled OLS and fixed effects.

F	DF1	DF2	p-Value
1.099	29	362	0.3342

**Table A7:** Performance comparison of polynomial features and the threshold value δ concerning the covariance value $\left(x_{i},y\right)\;$ on the target variable y=MDR−Pa.

m	*δ*	Selected Vars	R^2^ (Test)	RMSE (Test)	R^2^ (Train)	R_adjusted^2^ (Train)
3	0.5	68	0.392	0.094	0.778	0.718
3	0.3	111	0.637	0.073	0.876	0.810
4	0.4	83	0.222	0.107	0.805	0.737
4	0.6	34	−0.417	0.144	0.722	0.688
5	0.7	6	0.496	0.086	0.627	0.620
5	0.5	207	−7.278	0.349	0.931	0.803
2	0.3	11	0.510	0.085	0.662	0.650
2	0.2	19	0.558	0.080	0.705	0.686
2	0.1	33	0.567	0.079	0.730	0.699

**Table A8:** Performance comparison of polynomial features, type of algorithm, and the threshold value η on the target variable y=MDr−Pa.

m	Algorithm	*η*	Selected Vars	R^2^ (Test)	RMSE (Test)
3	LR	0.01	125	0.739	0.062
3	kNN	0.01	125	0.734	0.062
3	DT	0.01	125	0.538	0.082
3	LR	0.03	84	0.747	0.061
3	kNN	0.03	84	0.732	0.062
3	DT	0.03	84	0.563	0.080
3	LR	0.05	42	0.603	0.076
3	kNN	0.05	42	0.738	0.062
3	DT	0.05	42	0.381	0.095
2	LR	0.01	41	0.547	0.081
2	kNN	0.01	41	0.733	0.062
2	DT	0.01	41	0.608	0.076
2	LR	0.03	35	0.564	0.080
2	kNN	0.03	35	0.735	0.062
2	DT	0.03	35	0.527	0.083
5	LR	0.05	77	0.576	0.079
5	kNN	0.05	77	0.732	0.062
5	DT	0.05	77	0.406	0.093

LR: linear regression algorithm; KNN: k-nearest neighbors algorithm; DT: decision tree (DT) algorithm.

**Table A9:** Performance of the k-best variable selection and the recursive feature elimination methods on the target variable y=MDR−Pa.

m	Algorithm	Filter Method	Selected Vars	R^2^ (Test)	RMSE (Test)
3	LR	kBVS	60	0.653	0.071
3	kNN	kBVS	60	0.749	0.060
3	DT	kBVS	60	0.547	0.081
3	LR	kBVS	70	0.672	0.069
3	kNN	kBVS	70	0.759	0.059
3	DT	kBVS	70	0.669	0.069
3	LR	kBVS	80	0.699	0.066
3	kNN	kBVS	80	0.765	0.058
3	DT	kBVS	80	0.657	0.071
5	LR	kBVS	60	0.639	0.069
5	kNN	kBVS	60	0.713	0.065
5	DT	kBVS	60	0.671	0.077
3	LR	RFE	55	0.717	0.077
3	DT	RFE	7	0.662	0.084
5	LR	RFE	36	0.715	0.077
5	DT	RFE	64	0.716	0.077

LR: Linear regression algorithm; KNN: k-nearest neighbors algorithm; DT: decision tree (DT) algorithm; kBVS: k-best variable selection; RFE: recursive feature elimination.

**Table A10:** $R^{2}$ and root mean square error (RMSE) for the ML XGBoost and random forest algorithms models.

	Train (XGBoost)	Test (XGBoost)	Random Forest
$R^{2}$	0.92738	0.76828	0.80342
RMSE	0.03417	0.06279	0.05564
